# Primary Renal Embryonal Rhabdomyosarcoma in Adults: A Case Report and Review of the Literature

**DOI:** 10.1155/2012/460749

**Published:** 2012-10-23

**Authors:** Rafik Nabil Fanous, Erik K. Mayer, Justin Vale, Josephine Lloyd, Marjorie M. Walker

**Affiliations:** ^1^Department of Urology, St. Mary's Hospital, Imperial NHS Trust, London W2 1NY, UK; ^2^Department of Pathology, St. Mary's Hospital, Imperial NHS Trust, London W2 1NY, UK

## Abstract

Adult renal rhabdomyosarcoma is a rare subtype of renal sarcoma. We present a case of a renal mass treated with radical nephrectomy that subsequently was shown to be renal rhabdomyosarcoma. We discuss the clinical presentation, imaging findings, and histology for this case and review the available literature.

## 1. Introduction

Rhabdomyosarcomas of the kidney are a subtype of renal sarcomas which are rarely reported in the literature and arise from skeletal muscle progenitor cells. The histological subtypes include embryonal, alveolar, and pleomorphic variants. Primary rhabdomyosarcomas in the adult population are extremely rare. The majority of cases are seen in the paediatric population and are commonest in the head and neck region or urogenital tract. We report on a case of primary embryonal rhabdomyosarcoma (ERMS) of the kidney and review the available literature regarding diagnosis and management.

## 2. Case Report

A 37-year-old woman, of Middle Eastern descent, was admitted with a progressive six-month history of lethargy, weight loss, right flank pain, and macroscopic haematuria. She was passing thin “worms” of blood per urethra with abdominal discomfort on voiding. No significant past medical history was noted but she had a family history of liver carcinoma (paternal aunt).

Examination revealed a well female with stable vital signs. Respiratory and cardiovascular examination was unremarkable. The abdomen was mildly distended with a palpable ill-defined mass in the right upper quadrant found to be distinct from the liver. Abnormal laboratory investigations included a haemoglobin of 10.9 g/dL (11.5–16), haematocrit 0.322 (0.37–0.47), albumin of 30 g/L (35–51), C-reactive protein of 38 mg/L (0–5), and erythrocyte sedimentation rate of 93 mm/hr (<24). Serology revealed evidence of previous Epstein-Barr virus infection. Urine analysis was positive for blood (+++) and protein (+) with *Escherichia coli* cultured on a midstream specimen.

**Figure 1:**
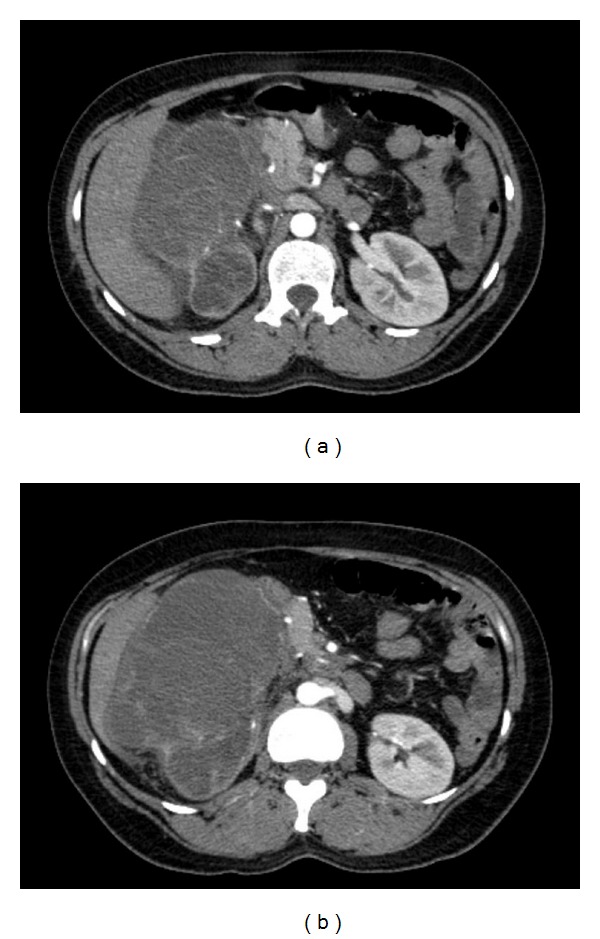
Cross-sectional computed tomography (CT) images showing a large heterogeneous mass replacing the right kidney.

Abdominal computed tomography (CT) demonstrated a large heterogeneous mass measuring 16 centimetres and replacing the right kidney (Figure 1). No definite local invasion was noted but the tumour was pushing on the liver, pancreas, and duodenum with no clear fat planes seen. CT did not show any thrombus in the inferior vena cava (IVC) above or below the tumour but it was reported that there was venous invasion of the right ovarian vein. Two enlarged lymph nodes were noted; however, there was no evidence of bony deposits or bowel pathology. By CT staging, this was at least T3, N1, and M0 with possible extension through the perinephric fascia inferiorly making this T4 disease.

The patient underwent an elective open right radical nephrectomy. At the time of surgery the tumour, originating from the right kidney, was partially necrotic and adherent to the liver and IVC. On further mobilisation of the tumour it was noted to extend into the right ureter. Tumour was identified to extend up to four centimetres above the bladder and the ureter was mobilised to below palpable disease and transfixed. The patient made an uneventful postoperative recovery and was discharged a week later. Repeated CT imaging at four months demonstrated no evidence of recurrent or metastatic disease.

Histology confirmed that almost the entire kidney was replaced by a multiloculated macrocystic and microcystic tumour measuring 145 × 140 × 95 mm. The tumour was partly solid with areas that were pale and homogenous and areas that were haemorrhagic. The ureter was filled with haemorrhagic and solid tumour similar in appearance to the solid haemorrhagic areas of the renal tumour. The tumour was predominantly spindle cell neoplasm with areas of small round blue cell tumour. Some cysts and epithelial components were noted at the renal pelvis; however, these were not thought to be part of the tumour and were thought to be secondarily involved. There was abundant haemorrhage and necrosis with an almost fibrinoid necrosis of vessels. The cells had variably scant and indistinct cytoplasm in most of the tumour although in some places eosinophilic cytoplasm was noted. Nuclei were varied in size but were mostly round or elongated.

Further immunohistochemical analysis revealed that the tumour cells expressed both Myogenin and focally MyoD1. There was strong staining in the cytoplasm for WT1 but relative sparing of the nucleus. The tumour was CD99 and PAX5 negative and lacked chromosomal changes associated with synovial sarcoma.

## 3. Discussion

Sarcoma of the kidney is rare and accounts for 1% of all primary renal malignancies [1, 2]. Of these, rhabdomyosarcomas are the least frequently reported in the literature. Adult cases are uncommon, arise mainly in large skeletal muscles, and are usually of pleomorphic subtype [3].

The classification of rhabdomyosarcomas was first described in 1958 into four histopathological subtypes: embryonal, botryoid-subtype of embryonal, alveolar, and pleomorphic [4, 5]. ERMS is the commonest subtype making up to approximately 66% of all diagnosed cases and has the best prognosis.

From the few cases in the literature it is difficult to make specific recommendations regarding diagnosis and management. It is clear, however, that ERMS behaves aggressively and presents late like other primary sarcomas. Median age of presentation for renal sarcomas is 49 years and the average size at diagnosis varies from 5.5 to 23 centimetres [6].

The diagnosis of primary renal sarcoma and specifically ERMS is difficult. The criteria for diagnosis of renal sarcomas includes three components as defined by Grignon et al. [7]. Firstly there should be no evidence of sarcoma elsewhere to exclude metastatic tumour from the differential. Secondly a sarcomatoid RCC must be excluded and this is by adequate sampling of the tumour to exclude an epithelial component. Finally, extension of a retroperitoneal sarcoma with secondary renal invasion can be excluded on histology.

Imaging characteristics of most sarcomas are indistinguishable from renal cell carcinoma and present as large nonspecific soft-tissue masses with poor contrast enhancement [6]. It is therefore difficult to offer any predictive features for ERMS on CT imaging. Thrombosis of the venous drainage including the IVC as identified on imaging can be useful for surgical planning [8]. In this case ovarian vein thrombosis was reported with no extension into the IVC. It is interesting to note that ureteric involvement was not picked up on CT. The nature of the surgery made it impossible to confirm or not if the ovarian vein was thrombosed or not and whether therefore if the appearances seen on CT corresponded to ureteric involvement instead. Whilst MRI was previously thought to be the gold standard for renal tumour imaging, several recent studies have shown comparable results with CT [9].

The diagnosis of ERMS is a difficult one to make on clinical and imaging findings with several differentials to consider. Histopathology after surgical resection is used to confirm the diagnosis, although this in itself can be challenging. Myogenin and MyoD1, myogenic regulatory proteins expressed early in skeletal muscle differentiation, are considered sensitive and specific markers for RMS and are more specific than desmin and more sensitive than myoglobin [10]. Mutation of the *WT1* gene is implicated in Wilm's tumours; however, in this case we found cytoplasmic staining with nucleus sparing. This does not support a diagnosis of Wilm's tumour and the literature suggests that this correlates with muscle differentiation and a diagnosis of RMS [11].

The literature is not sufficient to offer specific recommendations for treatment; however, it has been suggested that the treatment should be as for ERMS in any other site. The prognosis of primary renal sarcomas is poor with 90% of cases demonstrating metastases at the time of diagnosis [12]. The mainstay of treatment remains to be radical nephrectomy.

Studies in children have demonstrated a use for preoperative chemotherapy and postoperative radiotherapy. Evidence from the International RMS Study IV of 883 young patients (aged less than 20 years) showed improved survival in ERMS with three-drug chemotherapy [13]. There is much less data available for the use of chemotherapy and radiotherapy in adult cases.

Several studies have suggested that RMS in adults has a worse prognosis than in children. In contrast to the paediatric population, no association has been shown between survival and histological subtype in adults [14].

Data from a large series with a 20-year followup provides some insight into treatment options and prognosis. A total of 106 of 299 cases (35%) were rhabdomyosarcomas and of these only 16 (15%) were diagnosed in adulthood. No cases of primary renal disease were included. The data from this series demonstrates that whilst overall prognosis for genitourinary tumours across all ages is favourable (74% 5-year survival rate), this does not appear to hold true for adult disease. Only 1 of 6 adult genitourinary cases remained alive and disease free at the 10-month followup [15]. Several other studies have found the 5-year survival rate in adults to be around 35% [16].

In summary, primary ERMS is a rare entity in adults and there is little evidence to guide diagnosis and management in the literature. Whilst many suggest treatment as per paediatric protocols, there is growing evidence that the progression and prognosis of adult disease is unlike paediatric disease. Radical nephrectomy is the gold standard for treatment; however, there appears to be growing support for the use of neoadjuvant chemotherapy in long-term failure free survival.
